# Compassion and extracurricular activities of Portuguese Health Sciences students in Portugal

**DOI:** 10.1186/s12909-022-03419-2

**Published:** 2022-06-16

**Authors:** Luiz Miguel Santiago, Inês Rosendo, Catarina Valente, António Cruz Ferreira, José Augusto Simões

**Affiliations:** 1 Faculty of Medicine of the University of Coimbra, Coimbra, Portugal; 2 Clínica Universitária de Medicina Geral e Familiar da Faculdade de Medicina da Universidade de Coimbra, Coimbra, Portugal; 3 CEISUC, University of Coimbra, Coimbra, Portugal

**Keywords:** Compassion, Students, Health Sciences, Extracurricular Activities

## Abstract

**Abstract:**

**Background:**

Compassion, one of the items of empathy, is crucial in health care professions. So, the evaluation of the levels of compassion of Medicine, Dentistry and Pharmaceutical Sciences Master Degrees’ (M.D.) students of the public Colleges in Portugal according to the type of Master Degree and the participation in extracurricular activities (E.A.) was a task to be performed.

**Methods:**

Cross-sectional study in 2020, applying an on-line questionnaire including the “Compassion” items of the Jefferson Medical Empathy Scale – Students’ version and questions about the participation in E.A.

**Results:**

A sample of 901 students was studied. Its distribution by participation in E.A. did not differ significantly between M.D. (*p* = 0,854), most of the students participating in E.A. Using quartile distribution of compassion, the distribution of compassion levels was different among the three I.M. (*p* < 0.001), between Colleges (*p* < 0.001), and between curricular years (*p* < 0.001), with not different between genders (*p* = 0.036). For 56.4%, 74,6% and 69,5% of the respondents there was “medium-low” and “low” compassion for I.M. in Medicine, Pharmaceutical Sciences and Dentistry. These levels were also more prevalent among students in the 1st and 5th years. Levels of compassion were not different with the participation (*p* = 0,865), type (*p* = 0,177) and frequency of E.A. (*p* = 0,109).

**Conclusions:**

For their importance in future health care professionals, compassion and their differences found among the M.Ds. of this area deserve future studies. Levels of compassion showed differences between the M.D. studied and academic years of frequency. There was no relationship between the participation, type, and frequency of E.A. and the students’ levels of compassion.

The distribution of the level of compassion did not vary significantly with participation in E.A. (*p* = 0.865), with the type of E.A. (*p* = 0.177), with the frequency of E.A. (*p* = 0.109) or with the answer to the question “The practice of E.A. can make a person more compassionate?” (*p* = 0.503).

## Introduction

In Health Care practice, compassion is an essential part in the doctor-patient relationship. Compassion, is based on five elements: acknowledging the suffering of the other, understanding the universality of this suffering, adequate emotional response, tolerating difficulties and uncomfortable feelings that may be implicit (stress, anger and fear), and motivation to act, helping the person who is suffering [1, 2]. It has a positive impact on health care [3], providing an improvement in health out-comes and in the quality of information retained by the patient [1], as well as greater patient satisfaction with the services provided [1, 3] and less anxiety experienced [3]. As for physicians, compassionate behaviour is associated with greater job satisfaction [3].

Students in healthcare can present compassion as an innate quality. This quality can be maintained and even developed by educational reinforcements, in the context of clinical training and personal experiences, or even regressed [4]. Compassion, may also be described as one of the three components of empathy and be assessed in medical students [5, 6]. A previous study has adapted and validated the Jefferson scale for students in Portugal, so measuring Empathy as a whole and compassion in particular [6]. In brief Jefferson Scale of Physician Empathy – students’ Portuguese version (JSPE – spv) is a 20 items self-fulfilling questionnaire, each question rated 1 (strongly disagree) to 7 (strongly agree) intended to measure student’s perception about an empathic behaviour in the practical ambience of caring for a patient, and studying three dimensions: Perspective taking, compassion and being on the patient’s shoes [5]. Elsewhere and also in Portugal empathy and hence compassion have a negative trends in medical students as they progress in their studies, a suden decrease being noticed in the third year ane dependent on the Colleges teaching curriculum [5–9].

Medical students, compared to physicians, report more barriers to compassionate behaviour, particularly in the context of burnout [3]. A final work by a Medicine student proved the decline of students’ compassion throughout the Medicine Course at the College of Medicine of the University of Coimbra [7]. A work stemming from this, with the aim of investigating the main reasons for the decline of compassion in such training, gathered suggestions from students on tactics to implement to reduce the loss of compassion, suggesting the inclusion of volunteering and solidarity activities in the curriculum training and participation in extracurricular activities, among others [8]. To our knowledge the Jefferson scale for students is so far the only adapted and validated instrument for European spoken Portuguese and with published results, making it the only available choice for comparison within this country [5–9].

In the daily lives of medical students, the inclusion of extracurricular activities, with benefits such as establishing commitments, solving problems, and expressing emotions, can constitute a support for students to use, refine and develop their interpersonal skills [9, 10]. Extracurricular activities are also used as a strategy to deal with stress and burnout [10], and these activities are effective coping mechanisms in managing the stress to which students are subjected [11].

The aim of this study was to evaluate how the compassion of Integrated Masters students in the area of Health Sciences, from Public Schools in Portugal (Medicine, Pharmaceutical Sciences and Dentistry), was distributed according to the Masters and the practice of extracurricular activities as no paper on such objectives was ever published for this specific context in Portugal.

## Methods

A cross-sectional observational study was carried in a convenience sample of students from all curricular years of the three Integrated Masters in Health Sciences (Medicine, Pharmaceutical Sciences and Dentistry) from all Public Schools in Portugal (University of Minho, Porto, Coimbra, Covilhã, Lisbon, and Algarve) in a total of 14 schools in the 2019/2020 school year.

The population size was obtained taking into account the data available on the total number of students enrolled in the Integrated Masters in Medicine, Pharmaceutical Sciences and Dentistry in all public schools in the country and contained in the Data and Statistics page for Higher Education Courses, an official page from the Ministry of Sciences and Education [10] and the University of the Algarve’s page, DCBM-UAlg [11] the sample size being calculated for a confidence interval of 95% and a margin of error of 5%.

After approval by the Ethics Committee of the College of Medicine of the University of Coimbra, an online questionnaire was applied, organized into four parts:

A). Characterization data: Master’s and College, Curriculum year and gender.
B). The 7 items of the compassion dimension of the Jefferson Scale of Medical Empathy – Students Version [6]. For each item, the degree of agreement was requested from 1 (Strongly disagree) to 7 (Strongly agree). The Compassion dimension can have a maximum value of 49 points and a minimum value of 7 points. Each of his items is a negative sentence, the maximum value corresponding to a low level of Compassion. A quartile sorting work was carried out according to the numerical value of the “Compassion” indicator for better information on the topic, defining 4 categories: “High”, “Medium high”, “Medium low” and “Low”. A previous study has adapted and validated the Jefferson scale for students for Portugal, with a Cronbach’s alfa of 0.770 for the whole instrument and a 0,630 Cronbach’s alfa for “Compassion” [6].
C). Data related to Extracurricular Activities
  1. Participation in E.A.: Participation or non-participation.
  2. Type of main E.A. practiced: Physical/Individual Sports, Physical/Collective Sports, Cultural Activity (Theatre, Music, Arts...), Volunteering and Social Services, Associations and Paid Activity.
  3. Usual frequency of E.A..: once a week, twice a week, 3/4 times a week, 5/6 times a week, Every day, 1–3 times a month and Occasionally.
D). Affirmative or negative answer to a question regarding compassion: “Do you consider that the practice of Extracurricular Activity makes you more compassionate?”

The questionnaire was applied between October 2019 and January 2020, via a Google Forms. Students, course committees, student’s associations and communication departments were asked to share the “online” questionnaire with students at each College, using their Students social networks. The form explained the objective of the study and guaranteed anonymity, assuming the student’s consent to use the data for statistical treatment, based on their affirmative response to answer the questionnaire ticking a box allowing the questionnaire to be responded. No reminders were made.

Descriptive and inferential analysis was performed using SPSS Software for Windows version 24, after verifying the normality of the data. Analysis of the numerical variable of compassion value was performed as an ordinal variable depending on the function of the quartile distribution, with the lowest quartile being considered the one with the highest empathy.

Nonparametric Tests (Kolmogorov-Smirnov Test, Mann-Whitney Test, Kruskal Wallis Test) were used because of non-normal numeric data distribution and the Chi-Square Test were used, defining the difference as *p* < 0.001, due to the sample’s dimension.

## Results

In a universe of 15,045 students, the necessary sample size anticipated was of 375. A sample of *n* = 901 was obtained, 81.2% in IM of Medicine, 12.2% in Pharmaceutical Sciences and 6.5% in Dentistry. No answers were obtained from the College of Pharmaceutical Sciences of the University of Algarve. A Cronbach’s alfa of 0,726 for the chapter compassion of the Jefferson scale for students was obtained, if item deleted, the scoring ranging from 0,693 to 0,731.

By gender according to Table 1, a higher number of female responses (79.7%), a higher number of responses for 5th year students (20.1%) and a lower number of responses of the 1st year ones (12.5%) were found. Lisbon’s College of Medicine (24.0%) achieved the highest proportion of respondents.

**Table 1:** Characterization of the sample according to the Integrated Masters and gender, curricular year, and College

		Integrated Master			
		Medicine	Pharmaceutical Sciences	Dentistry	Total
		n (%)	n (%)	n (%)	n (%)
Gender^1^	Female	572 (78,1)	92 (83,6)	54 (91,5)	718 (79,7)
	Male	160 (21,9)	18 (16,4)	5 (8,5)	183 (20,3)
Curricular^2^ Year	1st year	75 (10,2)	38 (34,5)	0 (0,0)	113 (12,5)
	2nd year	105 (14,3)	13 (11,8)	1 (1,7)	119 (13,2)
	3rd year	141 (19,3)	23 (20,9)	8 (13,6)	172 (19,1)
	4th year	123 (16,8)	15 (13,6)	13 (22,0)	151 (16,8)
	5th year	123 (16,8)	21 (19,1)	37 (62,7)	181 (20,1)
	6th year	165 (22,5)	0 (0,0)	0 (0,0)	165 (18,3)
Total		732 (100)	110 (100)	59 (100)	901 (100,0)

^1^ *p* = 0,027; ^2^ *p* < 0,001 (Kruskal Wallis Test)

Also, according to Table 1 there was a significant difference in the distribution of students according to the Integrated Masters between the curricular years (*p* < 0.001), the different Colleges (*p* < 0.001). The distribution of students across the three Integrated Master’s Degrees did not differ significantly by gender (*p* = 0.027).

The distribution of participation in E.A. by the Integrated Masters, Table 2, reveals that such did not differ significantly among the studied I.M.(*p* = 0.854). An overall proportion of 76.7% of the respondents’ state to participate in E.A. There were no significant differences in the distribution of students in participation in E.A. between Colleges (*p* = 0.292), curricular years (*p* = 0.018) and gender (*p* = 0.089).

**Table 2:** Characterization of the sample according to the Integrated Masters and Participation in Extracurricular Activity

		Integrated Master			
		Medicine	Pharmaceutical Sciences	Dentistry	Total
		n (%)	n (%)	n (%)	n (%)
Participation in E.A.*	Yes	560 (76,5)	88 (80,0)	43 (72,9)	691 (76,7)
	No	172 (23,5)	22 (20,0)	16 (27,1)	210 (23,3)
Total		732 (100,0)	110 (100,0)	59 (100,0)	901 (100,0)

* *p* = 0,854 (Mann-Whitney U Test)

According to Table 3, the distribution of students by type of E.A. did not differ significantly between the three I.M. (*p* = 0.030), between Colleges (*p* = 0.543), between curricular years (*p* = 0.075), and between genders (*p* = 0.053).

**Table 3:** Characterization of the sample according to the Integrated Master’s Degree and Type of E.A. and Frequency of E.A

		Integrated Master			
		Medicine	Pharmaceutical Sciences	Dentistry	Total
		n (%)	n (%)	n (%)	n (%)
Type of EA^1^	Individual Physical/Sports	225 (40,2)	21 (23,9)	17 (39,5)	263 (38,1)
	Collective Physical/Sports	62 (11,1)	9 (10,2)	7 (16,3)	78 (11,3)
	Cultural Activity	95 (17,0)	14 (15,9)	6 (14,0)	115 (16,6)
	Volunteering and Social Services	102 (18,2)	24 (27,3)	3 (7,0)	129 (18,7)
	Community Associations	55 (9,8)	18 (20,5)	8 (18,6)	81 (11,7)
	Paid Activity	21 (3,8)	2 (2,3)	2 (4,7)	25 (3,6)
Frequency of EA^2^	Occasionally	35 (6,3)	4 (4,5)	1 (2,3)	40 (5,8)
	1–3 times a month	19 (3,4)	7 (8,0)	1 (2,3)	27 (3,9)
	1 time a week	80 (14,3)	22 (25,0)	3 (7,0)	105 (15,2)
	2 times a week	170 (30,4)	26 (29,5)	21 (48,8)	217 (31,4)
	3/4 times a week	183 (32,7)	16 (18,2)	12 (27,9)	211 (30,5)
	5/6 times a week	34 (6,1)	4 (4,5)	3 (7,0)	41 (5,9)
	Every day	39 (7,0)	9 (10,2)	2 (4,7)	50 (7,2)
Total		560 (100)	88 (100)	43 (100)	691 (100,0)

^1^ *p* = 0,030; ^2^ *p* = 0,131 (Kruskal Wallis Tests)

According to the distribution of the type of E.A. by the I.M. (Table 3):

- The activity “Individual Physical/Sports” was the most practiced by students in general (38.1%), followed by “Volunteering and Social Services” (18.7%).
- Among medical students, the activity “Individual Physical/Sports” was the most practiced (40.2%), followed by “Volunteering and Social Services” (18.2%).
- In Pharmaceutical Sciences students, “Volunteering and Social Services” was the most reported activity (27.3%), followed by “Individual Physical/Sports” (23.9%).
- Among Dentistry students, the activity “Individual Physical/Sports” (39.5%) was the most practiced, followed by Community Associations (18.6%).
- Paid Activity was the least reported among students (3.6%).

The frequency of students by E.A. type did not differ significantly between I.M. (*p* = 0.131), between Colleges (*p* = 0.435), between curricular years (*p* = 0.161), and genders (*p* = 0.021), with the participation of twice a week being more reported (31.4%), followed by 3/4 times a week (30.5%) (Table 3).

According to Table 4 the results of the Jefferson Scale of Medical Empathy – Students Version ranged between 7 and 34, with a mean of 14.36 ± 4.81 and a median of 13.

**Table 4:** Levels of compassion as a function of quartile distribution according to Integrated Master’s Degree and year attended

	Levels of compassion				
	High	Medium-High	Medium-Low	Low	Total
	n (%)	n (%)	n (%)	n (%)	n (%)
Integrated Master *					
Medicine	182 (24,9)	137 (18,7)	225 (30,7)	188 (25,7)	732 (100,0)
Pharmaceutical Sciences	13 (11,8)	15 (13,6)	30 (27,3)	52 (47,3)	110 (100,0)
Dentistry	10 (16,9)	8 (13,6)	20 (33,9)	21 (35,6)	59 (100,0)
Year **					
1st year	13 (11,5)	19 (16,8)	35 (31,0)	46 (40,7)	113 (100,0)
2nd year	21 (17,6)	16 (13,4)	48 (40,3)	34 (28,6)	119 (100,0)
3rd year	37 (21,5)	36 (20,9)	51 (29,7)	48 (27,9)	172 (100,0)
4th year	42 (27,8)	26 (17,2)	43 (28,5)	40 (26,5)	151 (100,0)
5th year	42 (23,2)	33 (18,2)	44 (24,3)	62 (34,3)	181 (100,0)
6th year	50 (30,3)	30 (18,2)	54 (32,7)	31 (18,8)	165 (100,0)
Gender ***					
Female	167 (81,5)	137 (85,6)	220 (80,2)	194 (74,3)	718 (79,7)
Male	38 (18,5)	23 (14,4)	55 (20,0)	67 (2578)	183 (20,3)
Total	205 (22,8)	160 (17,8)	275 (30,5)	261 (29,0)	901 (100,0)

* *p* < 0,001 (Kruskal Wallis Test); **p = 0,005(Kruskal Wallis Test);***p = 0,016 (Mann-Whitney U Test)

According to the quartile distribution of compassion, the most frequent level is the “Medium-Low” (30.5%), followed by the “Low” level (29.0%).

The distribution of compassion levels was different among the three I.M. (*p* < 0.001), between Colleges (*p* < 0.001), and between curricular years (*p* < 0.001), with no differences in the levels of compassion by gender (*p* = 0.036). The distribution of the level of compassion did not vary significantly with participation in E.A. (*p* = 0.865), the type of E.A. (*p* = 0.177), the frequency of E.A. (*p* = 0.109) or with the answer to the question “The practice of E.A. can make a person more compassionate?” (*p* = 0.503). A week negative, yet significant correlation was found between the Curricular Years of College attendance and the Quartile level of compassion (ρ:-0.131; *p* < 0.001).

The distribution of levels of compassion across the three I.M. shows that:

- In Medicine, the level “Medium-Low” was more frequent (30.7%) and the result “Medium High” (18.7%) was less frequent.
- In Pharmaceutical Sciences, the most frequent level was “Low” (47.3%) and the least frequent was “High” (11.8%).
- In Dentistry, the “Low” level was more frequent (35.6%) and “Medium High” was the least frequent (13.6%).

In the three I.M., the two most frequent levels were “Medium-Low” and “Low”, with 56.4% of Medicine students, 74.6% of Pharmaceutical Science students, and 69.5% of Dentistry students showing levels of compassion from “Medium-Low” to “Low”.

At the levels of compassion according to the I.M. and the curricular year, “Low” levels of compassion were more frequent among students in the 1st and 5th years (40.7 and 34.3% respectively).

In 2nd, 3rd, 4th, and 6th year students, “Medium-Low” levels of compassion were more frequent (40.3, 29.7, 28.5, and 32.7% respectively).

## Discussion

This study, carried out in 2019, addressed, for the first time in Portugal, the relationship between the levels of compassion of I.M. in health areas and the practice of E.A. using the Jefferson Scale of Medical Empathy – Students Version, adapted for Portugal [6].

Most students participated in E.A. (76.7%), in a higher proportion than previously reported [9, 12] with a preference for the “Individual Physical/Sports” activity with 49.4%, a lower value than previously reported [11, 12]. “Volunteering and Social Services” was reported by (18.7%), obtained a value higher than that already observed for medical students [11], but lower than that already observed in Dentistry and Pharmaceutical Sciences [12, 13]. Remunerated Activities continue to be the least mentioned [11]. Thus, the observed structure will probably bring changes in the future population of IM students with probable practice patterns changes, which must be studied and perceived. And Colleges should be attentive to this phenomenon.

Compassion scale answer results are worrisome with a higher proportion of “Medium-Low” and “Low” especially for the I.M Pharmaceutical Sciences students. A study of the Master’s Degree of Pharmacy at Queen’s University of Belfast reported that students who did not experience clinical contact with patients as extensive as students of Dentistry and Medicine [14], and this was a factor for less compassion which could justify the present levels. And in fact, the vast majority of Pharmaceutical Sciences will deal with the real world persons.

Studies that used the Jefferson Scale of Medical Empathy [5, 6], revealed contradictory results regarding the distribution and evolution of compassion and empathy over the curricular years of Medicine, Pharmaceutical Sciences, and Dentistry [4, 7, 15–23]. We found that the 1st year and the 5th year are those with the highest percentage of “Low” compassion levels, with the 5th year being the last year of the I.M. of Pharmaceutical Sciences and Dentistry. It is therefore relevant to come to an understanding of the reasons for the prevalence of lower levels of compassion among final year students of Pharmaceutical Sciences and Dentistry, soon to be health professionals.

In a Portuguese study on medical students, it was reported that the formal curriculum and various factors such as “...limited time of contact with patients”, “...academic training essentially promoting theoretical/organic knowledge and the search for diagnosis”, “...lack of training to deal with psychological/emotional aspects”, “…excess of workload inherent to the curriculum” were considered to justify the decline of compassion throughout medical training [8]. A later study, also with medical students, found that the difference in the levels of compassion among students from the various Portuguese Colleges of Medicine studied did not depend on the percentage of the European Credit Transfer and Accumulation System in the curriculum, related to the compassion approach [15]. It will be pertinent to study if these and other factors also affect Pharmaceutical Sciences and Dentistry students.

In the present study, there were no significant differences in compassion between genders, unlike other studies [16–18]. Present results are in-line with those described for Pharmacy students in the United Kingdom and with the ones in 1st year medicine students in Portugal [14, 20].

The present study found no relationship between extracurricular activities and the students’ levels of compassion, even though 93.7% considered that “the practice of E.A. can make a person more compassionate”, which a Portuguese study had suggested, as a tactic to reduce the decline of compassion throughout the I.M. in Medicine [7, 9]. So an assumption can be made that there is a will but not a way, deserving future studies in the Portuguese context. In fact one may assume that this is indeed a wishful thinking students believe in but are not willing to perform maybe because of curricular obligations, tasks and professional future to think about.

Studies report that students participation in E.A. can improve teamwork skills, networking, better time management, helping others and contact with people of different perspectives, acquiring new knowledge, and expanding the individual curriculum [9, 12, 13]. Reports also exist stating that improvement of stress management capacity was indicated as an effective coping mechanism in stress management [11], physical activities being related to low levels of stress [10], and Volunteering and Social Services promoting solidarity, social responsibility, and a sense of community [10]. And it was also described that Community Associations, involving organization and leadership, are associated with better academic performance [11]. Due to the named advantages, encouragement by the Universities/Colleges was suggested, enhancing the reasons for the participation of students in E.A. and eliminating impediments, somehow integrating them into the formal curriculum [9]. Physical activities, were the most reported E.A., the need to study if an Individual or Team one is more prone to maintain or increase compassion, now being a future question of study. So it is possible that policy changes in Portuguese colleges can bring about better results.

The present study has some limitations namely having been carried out in a convenience sample, although representative of the quantitative universe of students, thus preventing the generalization of the results and the possibility of social desirability bias associated with the use of Likert-type scales, even with the guarantee of confidentiality and anonymity. The Questionnaire appliance between October 2019 and January 2020, with no reminders, was long enough so that different times of the school year could have resulted in different responses, for two different semesters were enrolled. The lack of knowledge about the teaching that each School does about Compassion, as well as the particular characteristics of each respondent, must also be considered when reading these results.

Despite the limitations, the present study alloww to retain considerations about the distribution of levels of compassion among students of Integrated Masters in Health Sciences as a result of the practice of extracurricular activities. Further work on analysing the reasons for the differences found and designing measures to improve compassion, that can be taught and worked on, should now follow.

## Conclusions

There were differences in the distribution of levels of compassion between the three I.M., and between the curricular years. Most students of each I.M. showed levels of compassion from “Medium-Low” to “Low”, revealing the Pharmaceutical Sciences as having the highest percentage of students with lower levels, followed by I.M. in Dentistry.

“Low” levels of compassion were also more prevalent in 1st and 5th-year curricular students, as well as in males.

There was no evidence of a relationship between participation, type, and frequency of extracurricular activities and students’ levels of compassion.

## Data Availability

The datasets used and/or analysed during the current study available from the corresponding author on reasonable request.

## Abbreviations

E.A.: Extracurricular Activities
I.M.: Integrated Master
